# Space weathering signatures in sulfide and silicate minerals from asteroid Itokawa

**DOI:** 10.1186/s40623-022-01683-6

**Published:** 2022-08-09

**Authors:** Laura C. Chaves, Michelle S. Thompson

**Affiliations:** Department of Earth, Atmospheric, and Planetary Sciences, 550 Stadium Mall Drive, West Lafayette, IN 47907 USA

**Keywords:** Hayabusa, Itokawa, Space weathering, Sulfides, Transmission electron microscopy, Energy-dispersive X-ray spectroscopy

## Abstract

Transmission electron microscopy analyses of the polymineralic regolith particle RC-MD01-0025 show microstructural and microchemical characteristics indicative of space weathering on the surface of asteroid Itokawa. The depletion of sulfur and nickel was identified in space weathered rims on troilite and pentlandite minerals. This corresponds to the first report of nickel depletion in samples returned from asteroid Itokawa by the Hayabusa mission. Microstructurally, the sulfide minerals present crystalline rims and the olivine presents both crystalline and amorphous zones in the rim. These results suggest that sulfides might be more resistant to amorphization caused by solar wind irradiation. The space weathering features identified in the regolith particle analyzed here are likely formed via solar wind irradiation. Additionally, the differences in the space weathering features in olivine, pentlandite, and troilite suggest that silicates and sulfides respond differently to the same space weathering conditions in interplanetary space.

**Graphical Abstract:**

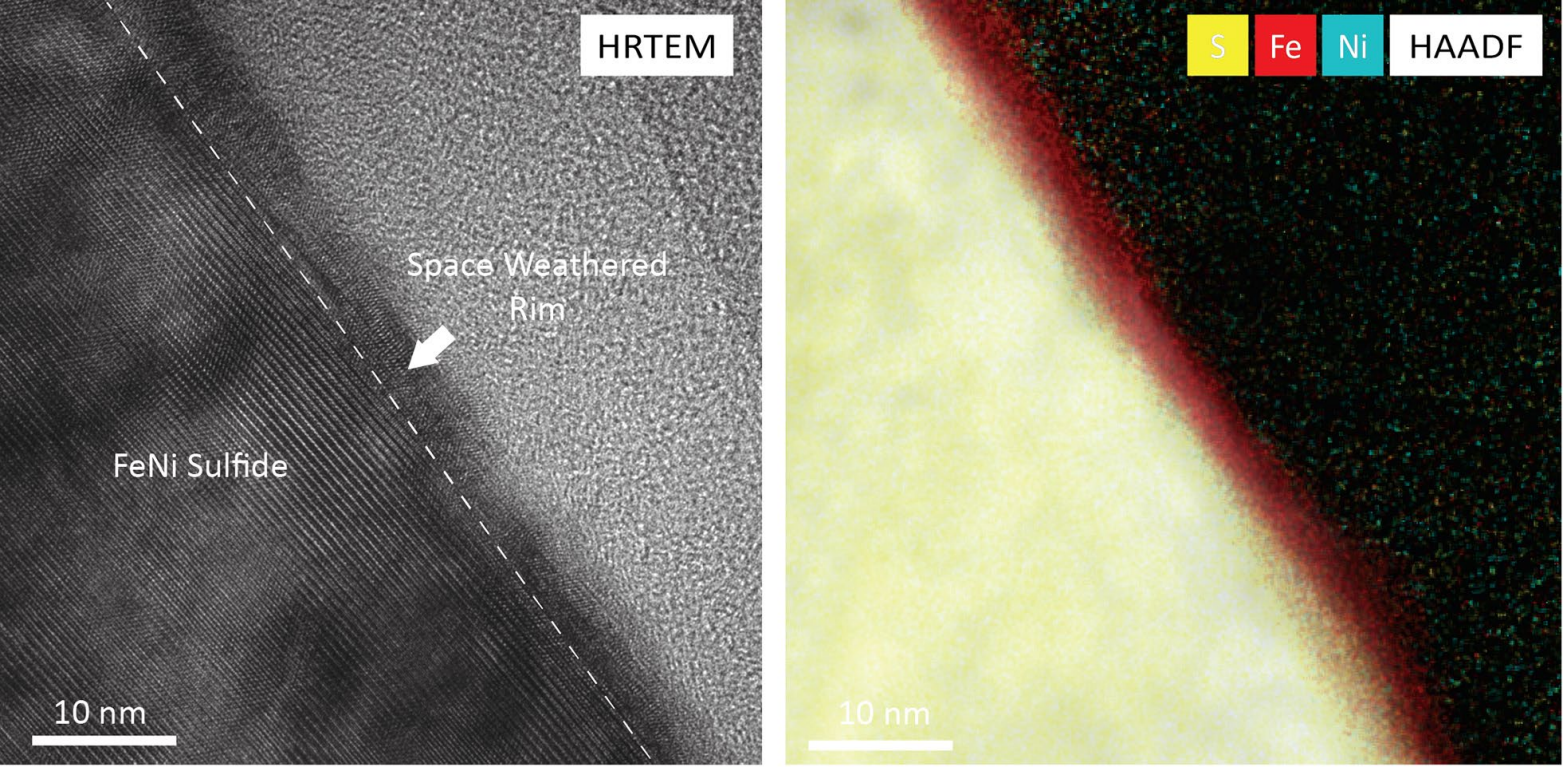

## Introduction

In 2010, the Japan Aerospace Exploration Agency’s (JAXA) Hayabusa mission returned 1534 regolith particles to Earth from the S-type asteroid 25143 Itokawa (Nakamura et al. 2011). S-type asteroids were long-hypothesized to be the parent bodies for ordinary chondrite meteorites (Chapman 2004). However, the reflectance spectra of S-type asteroids exhibit a redder slope across the visible to near-infrared (VNIR) wavelengths and attenuated absorption features compared to the ordinary chondrite meteorites in our collections. In advance of sample return from the Hayabusa mission, this spectral dissimilarity was attributed to space weathering of asteroidal surfaces, which their meteorite counterparts did not experience (Binzel et al. 1996). Petrological analysis of regolith particles collected by the Hayabusa mission showed a composition consistent with LL4-6 chondrites, thus confirming the link between ordinary chondrites and S-type asteroids. This relationship between ordinary chondrites and S-type asteroids confirmed by the Hayabusa mission provides evidence of the importance of understanding space weathering processes in order to accurately characterize the composition of airless surfaces and link meteorites to their parent bodies.

As demonstrated by the results of the Hayabusa mission thus far, space weathering alters the chemical, microstructural, and spectral properties of regolith particles on the surfaces of airless bodies and is driven primarily by micrometeoroid impacts and solar wind irradiation (Hapke 2001; Pieters and Noble 2016). Historically, our understanding of space weathering has been based on lunar samples collected during the Apollo missions. In lunar soils, we observe the attenuation of characteristic absorption bands, reddening, and darkening of reflectance spectra when compared to lunar samples that were unexposed to interplanetary space (e.g., rock interiors). The spectral anomalies in lunar soils are largely attributed to the presence of iron nanoparticles, predominantly composed of metallic iron (npFe^0^) (Keller et al. 1998; Hapke 2001). In addition to spectral changes, space weathering also causes the development of microstructural and chemical characteristics in lunar soil particles at the nanoscale including vapor and melt deposits, amorphous rims, and iron nanoparticles (Keller and McKay 1993, 1997; Burgess and Stroud 2018; Gillis-Davis et al. 2020).

Several experimental efforts have been made to understand the individual contributions of micrometeoroid bombardment and solar wind irradiation to the space weathering of airless bodies. Pulsed-laser irradiation has been employed to simulate the short-duration, high temperature events associated with hyper-velocity micrometeoroid bombardment (Yamada et al. 1999; Sasaki et al. 2001; Loeffler et al. 2016; Fazio et al. 2018; Thompson et al. 2019). Ion irradiation with 1 keV H^+^ and 4 keV He^+^ has been used to simulate the major constituents of the solar wind (Dukes et al. 1999; Brunetto and Strazzulla 2005; Loeffler et al. 2009; Lantz et al. 2017; Laczniak et al. 2021). Each of these experimental techniques have produced spectral, microstructural, and chemical characteristics consistent with space weathering on airless bodies. However, most laboratory simulations and studies of returned samples have focused on understanding the effects of space weathering processes on silicate minerals which dominate lunar soils and ordinary chondrites (Keller and McKay 1993, 1997; Dukes et al. 1999; Sasaki et al. 2001; Kohout et al. 2014; Loeffler et al. 2016). Meanwhile, the situation is less constrained for minerals such as Fe- and Fe–Ni-sulfides that are found on other airless planetary bodies including ordinary chondritic asteroids like Itokawa (Keller and Berger 2014; Matsumoto et al. 2020; Burgess and Stroud 2021), in carbonaceous materials like those returned from asteroid Ryugu (Han et al. 2022; Matsumoto et al. 2022; Viennet et al. 2022) and those expected from asteroid Bennu (Hamilton et al. 2019), and in CB chondrites the closest meteoritic counterpart for Psyche (Weisberg et al. 2001; Elkins-Tanton et al. 2020).

Similar to investigations of lunar soils, analysis of the space weathering characteristics of Itokawa grains has predominantly focused on silicate minerals, including pyroxene, olivine, and plagioclase. Those analyses have revealed completely and partially amorphous rims, multilayer rims that are chemically distinct from the underlying grain, vesiculated textures, and Fe- and Fe-sulfide nanoparticles (npFeS) (Noguchi et al. 2011, 2014a, b; Keller and Berger 2014; Thompson et al. 2014; Burgess and Stroud 2021). In particular, the presence of npFeS in Itokawa samples was novel, as the nanoparticles in space-weathered lunar samples are predominantly composed of Fe^0^ (Keller and McKay 1993; Thompson et al. 2016; Burgess and Stroud 2018). The identification of FeS nanoparticles suggests that sulfide minerals might play a critical role in the space weathering of asteroidal surfaces (Keller et al. 2013).

Recent investigations of the space weathering of sulfide minerals have indicated these phases respond in new ways to surface processes on airless bodies. Analyses of Fe-sulfides in both Itokawa grains and lunar soils have revealed Fe whiskers protruding from sulfide grain surfaces (Matsumoto et al. 2020, 2021). The formation of these whisker structures is attributed to a combination of preferential sputtering and depletion of S in the outer grain rims and supersaturation of Fe^2+^ as a consequence of space weathering and the subsequent reduction of Fe^2+^ to Fe^0^ by free electrons on the sulfide surfaces (Matsumoto et al. 2021). Additionally, Keller and Berger (2014) observed similar evidence of this sulfur depletion and the occurrence of Fe^0^ nanoparticles in a disordered rim in an Itokawa pyrrhotite-bearing particle.

The sulfur depletion evidenced in Hayabusa samples has also been observed in laboratory simulations of space weathering processes. Loeffler et al. (2008) performed both ion and pulsed-laser irradiation experiments to replicate solar wind irradiation and micrometeoroid impacts on troilite (FeS) powders. These irradiation experiments revealed segregation of sulfur to the surface of the troilite following the pulsed-laser irradiation experiments. In contrast, the 4 keV He^+^ and 4 keV Ar^+^ irradiation experiments resulted in a decrease in the S:Fe ratio of troilite powders, suggesting constituent space weathering processes may have competing effects on the retention of sulfur in sulfide minerals exposed to space weathering on airless surfaces. In addition to the microstructural and chemical characterization of laboratory space weathered sulfides, previous attempts have been made to understand the spectral effects of micrometeoroid impacts in sulfide minerals. Prince et al. (2020) identified an initial increase in the reflectance, followed by reduction of reflectance and spectral slope of the VNIR spectra of troilite as result of pulsed-laser irradiation experiments. These results suggest that alteration of sulfide minerals under space weathering conditions is unique and might contribute significantly to changing the spectral properties of airless bodies.

Laboratory simulations and analysis of returned samples are also crucial to the interpretation of remote sensing observations. Sulfur depletion has been measured in sulfide-bearing Itokawa regolith particles (Keller and Berger 2014; Burgess and Stroud 2021), but was not identified in the X-ray fluorescence spectrometry (XRF) analyses of the surface of asteroid Itokawa (Arai et al. 2008). However, the XRF data show a global variation of S that could result from localized space weathering processes (Arai et al. 2008). While a global sulfur depletion was not observed spectrally for Itokawa, a low S/Si ratio was identified in the XRF data for asteroid 433 Eros (Nittler et al. 2001). These results suggest that the processing of sulfur on asteroidal surfaces is complex and demonstrates a need for improved understanding of the response of S-bearing minerals to space weathering conditions. To advance our knowledge of sulfide mineral space weathering, here we report transmission electron microscopy (TEM) analyses of a sulfide-bearing Itokawa regolith particle to better understand the response of Fe- and Fe–Ni sulfides to space weathering conditions. In this study, we compare the microstructural and chemical signatures of silicates and sulfides that could be attributed to space weathering. Understanding the response of sulfide minerals to interplanetary space through the analysis of Itokawa regolith particles is essential for interpreting remote sensing data from asteroids Itokawa and Psyche, as well as for maximizing the science return for the Hayabusa, Hayabusa2, and OSIRIS-REx missions.

## Methods

We studied the RC-MD01-0025 Itokawa regolith particle that was previously identified to contain low-Ca pyroxene, olivine, plagioclase, and Fe- and Fe–Ni-sulfide minerals by energy-dispersive X-ray spectroscopy (EDS) analysis in the scanning electron microscope (SEM) by the JAXA curation team before allocation. The regolith particle is angular in shape and has approximate dimensions of 31 µm in length by 17 µm of width. The particle was embedded in low-viscosity epoxy and ultramicrotomed to prepare electron transparent thin sections (~ 50 nm thick) that were transferred to C-coated Au grids for TEM analysis. The particle sections were studied using a 200 keV FEI Talos TEM at Purdue University. The sample was analyzed using both conventional TEM and scanning TEM (STEM) techniques, including high-resolution TEM (HRTEM), as well as bright field (BF) and high-angle annular dark field (HAADF) images to identify space weathering characteristics in the particle. EDS maps and linescans were acquired using a Super-X silicon drift detector on the Talos in order to identify the phases present in the regolith particle and the chemical characteristics of space weathering. Quantitative chemical data were obtained using the Cliff–Lorimer method, and we used dwell times of 50 µs, with count rates between 5 and 35 kcps, and a probe size < 1 nm.

## Results

### Pentlandite

**Fig. 1:**
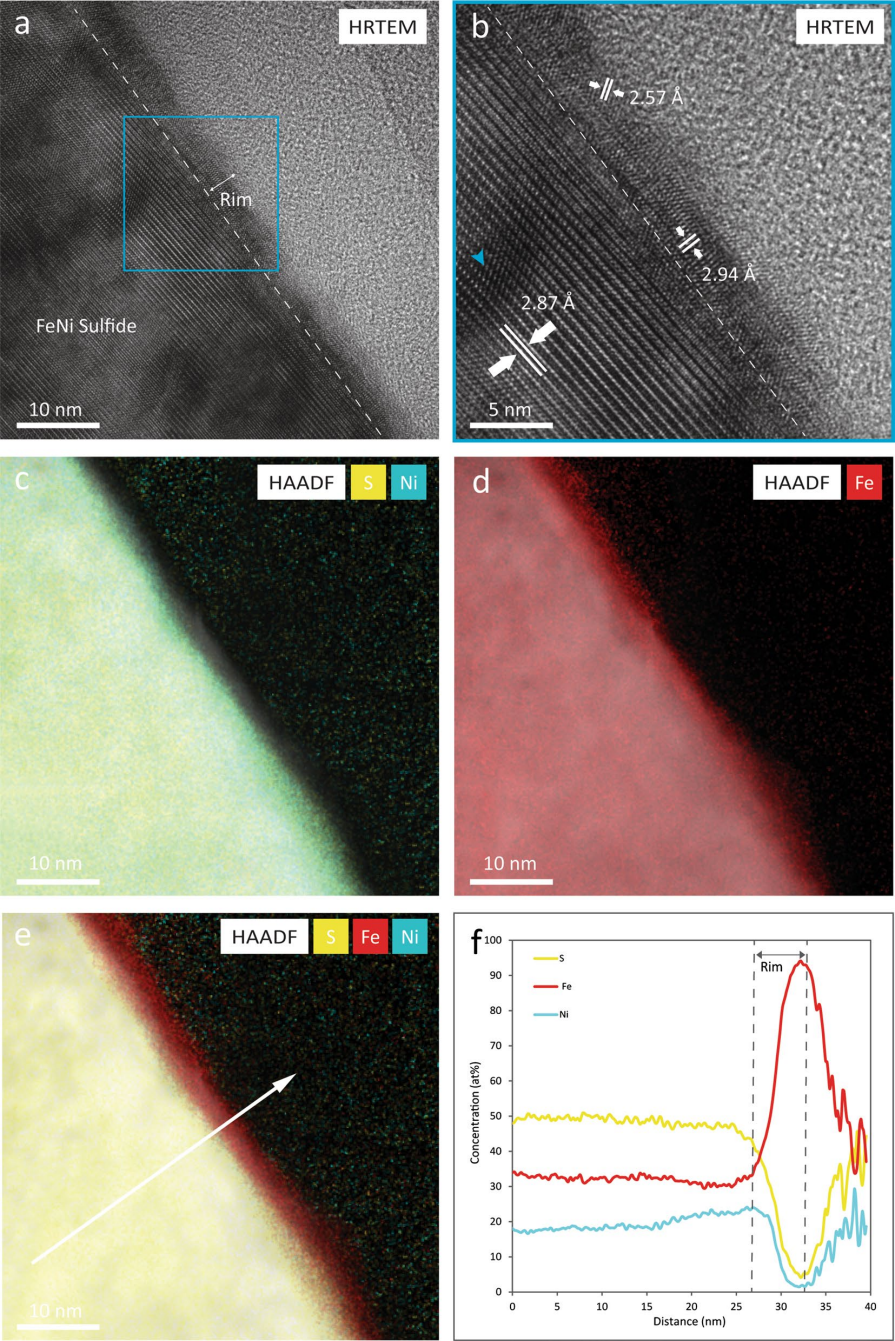
HRTEM and EDS chemical mapping of Fe-Ni-sulfide grain. **a** HRTEM image showing the presence of a crystalline rim in the outer ~ 5–10 nm of the sulfide grain. **b** HRTEM image revealing lattice fringes with d-spacing values of 2.87 Å in the interior of the grain similar to (222) pentlandite and 2.94 Å and 2.57 Å in the rim consistent to d-spacing values for pentlandite, hematite, and magnetite. In addition, blue arrow shows the presence of strained regions in the grain. **c** HAADF image overlain with EDS maps of S and Ni. **d** HAADF image with Fe EDS map. **e** HAADF image with EDS maps of S, Ni, Fe showing the location of the line scan. **f** Quantitative EDS line scan indicating depletion of S and Ni, and the enrichment of Fe in the crystalline rim

EDS chemical analyses of the Fe–Ni-sulfide show a normalized chemical composition (at%) of Fe (35.0), Ni (14.0), and S (51.0) similar to the Fe, Ni, and S compositions measured in pentlandite grains from LL chondrites by Schrader et al. (2016) and Schrader and Zega (2019). High-resolution transmission electron microscopy (HRTEM) shows evidence of a crystalline rim in the outer 5–10 nm of the grain surface (Fig. 1a). Additionally, HRTEM imaging reveals that the interior of this grain exhibits lattice fringes with d-spacing values of 2.87 Å, similar to (222) pentlandite. The crystalline rim presents lattice fringes with d-spacing values of 2.94 Å and 2.57 Å that are difficult to attribute it to a single mineral phase as these values are similar to d-spacing values for pentlandite and for various Fe oxide minerals including hematite and magnetite (Fig. 1b). Additionally, strained regions were identified in the pentlandite grain as shown by the blue arrow in Fig. 1b. EDS mapping of the grain rim indicates that the crystalline rim is depleted in S and Ni but enriched in Fe (Fig. 1c, d). To quantify the chemical differences between the interior pentlandite grain and the crystalline rim, line scans were acquired to determine variations in Fe, Ni, and S (Fig. 1e, f). The normalized concentration values of the interior of the grain are 48 at% S, 32 at% Fe, and 20 at% Ni. In the rim, the values are 18 at% S, 72 at% Fe, and 10 at% Ni. To summarize, the rim presents a depletion of S of 30 at% and Ni of 10 at%, and an enrichment of Fe of 40 at% compared to the interior. In addition, O was identified in the rim of the grain, attributed to oxidation from exposure to atmosphere.

### Troilite

**Fig. 2:**
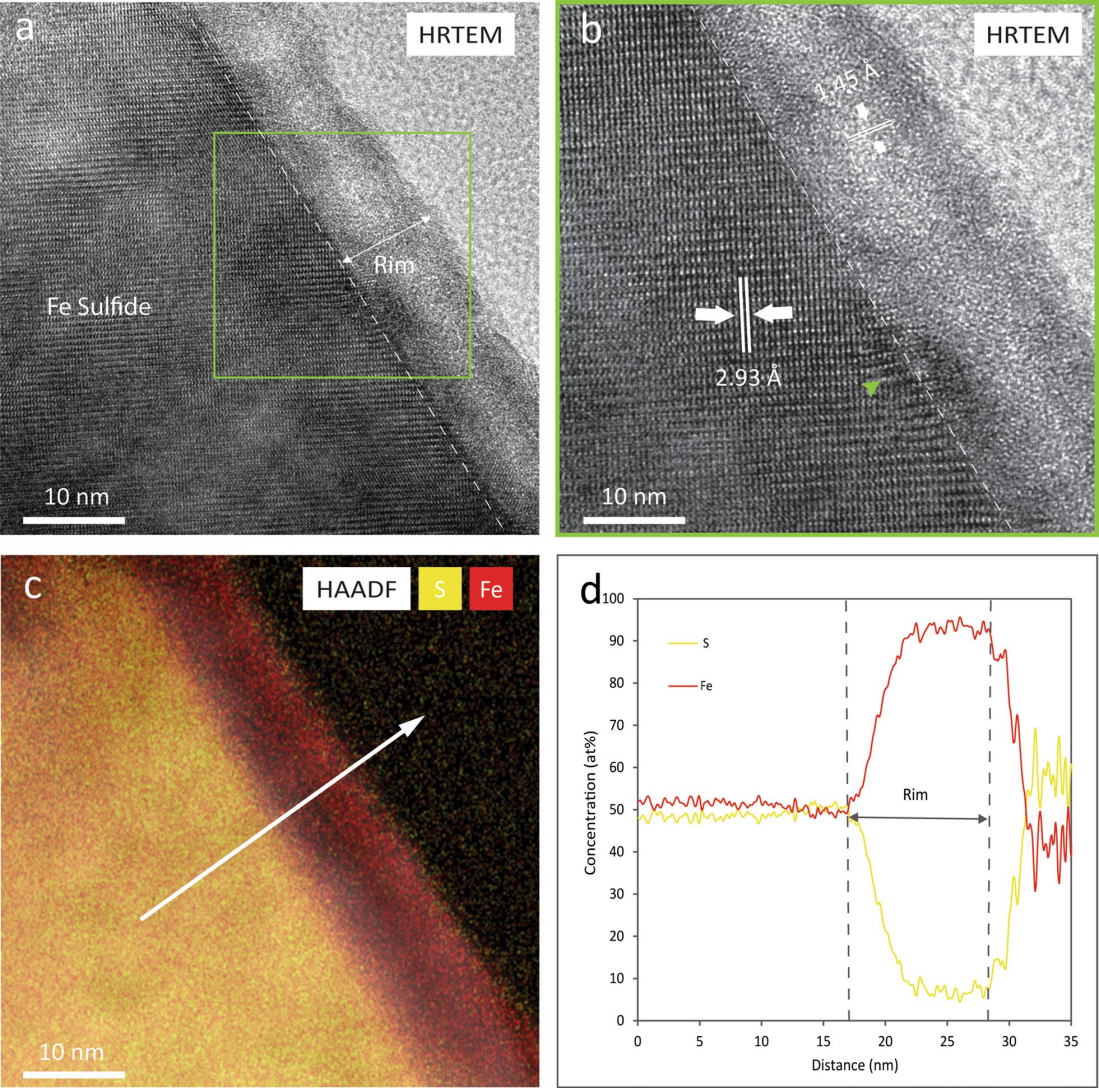
Microstructural and chemical properties of space weathered Fe-sulfide. **a** HRTEM images showing the occurrence of a nanocrystalline rim in the outermost ~ 10 nm of the grain. **b** HRTEM image indicating that the unaltered region presents d-spacing value of 2.93 Å that matches (110) troilite and the rim has lattice fringes with values of 1.45 Å similar to (200) metallic iron. A strained region is shown by the green arrow. **c** HAADF image overlain with S and Fe EDS maps and **d** EDS line scan revealing that the nanocrystalline rim is depleted in S and enriched in Fe compared to the interior of the grain

A normalized composition of Fe (49.6 at%), S (50.4 at%), and an average at% Fe/S ratio of 0.98 were obtained by quantitative EDS analyses. These compositional values and Fe/S ratios are similar to values reported for troilite in LL chondrites (Schrader and Zega 2019; Schrader et al. 2021). HRTEM imaging of the troilite grain reveals the occurrence of a nanocrystalline rim in the outer ~ 10 nm (Fig. 2a) of the grain surface. In addition, HRTEM indicates that the interior of the troilite grain presents lattice fringes with a d-spacing value of 2.93 Å similar to (004) troilite whereas the rim has d-spacing value of 1.45 Å consistent to (200) metallic iron (Fig. 2b). Moreover, strained regions were identified in the interior of grain/rim interphase as shown by the green arrow in Fig. 2b. The chemical analysis of the nanocrystalline troilite rim shows that the rim is enriched in Fe and depleted in S compared to the interior of the grain, evidenced by the EDS map and quantitative line scan (Fig. 2c, d). The values from the interior of the grain are 49 at% S and 51 at% Fe, and in the rim the values are 17 at% S and 83 at% Fe. This results in a depletion of S of 32 at % and an enrichment of Fe of 32 at% in the rim relative to the interior. As in the pentlandite grain, we identified O in the nanocrystalline rim. Oxidized rims on sulfides from asteroid Itokawa have been reported previously by Burgess and Stroud (2021). The oxidation of sulfides was most likely produced by the exposure of the regolith particle to Earth’s atmosphere during sample preparation.

### Olivine

**Fig. 3:**
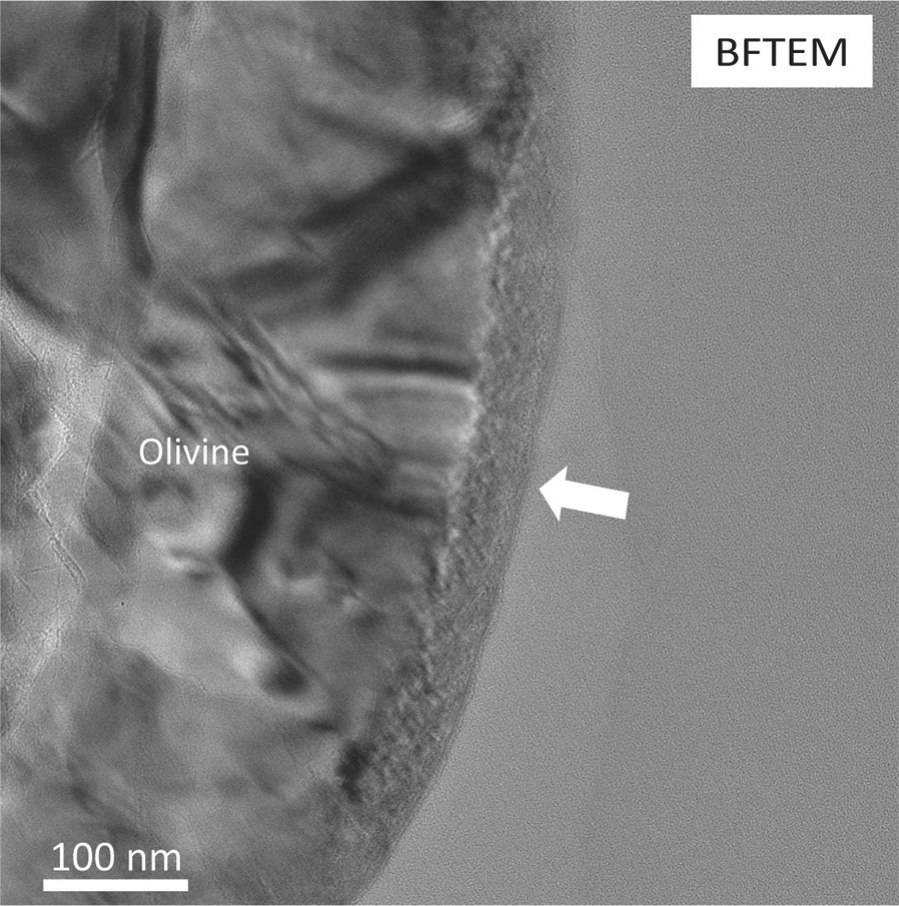
Space weathering rim on the olivine grain. BFTEM image showing the presence of a discontinuous rim with an approximate thickness of 50 nm in the surface of the olivine grain

**Fig. 4:**
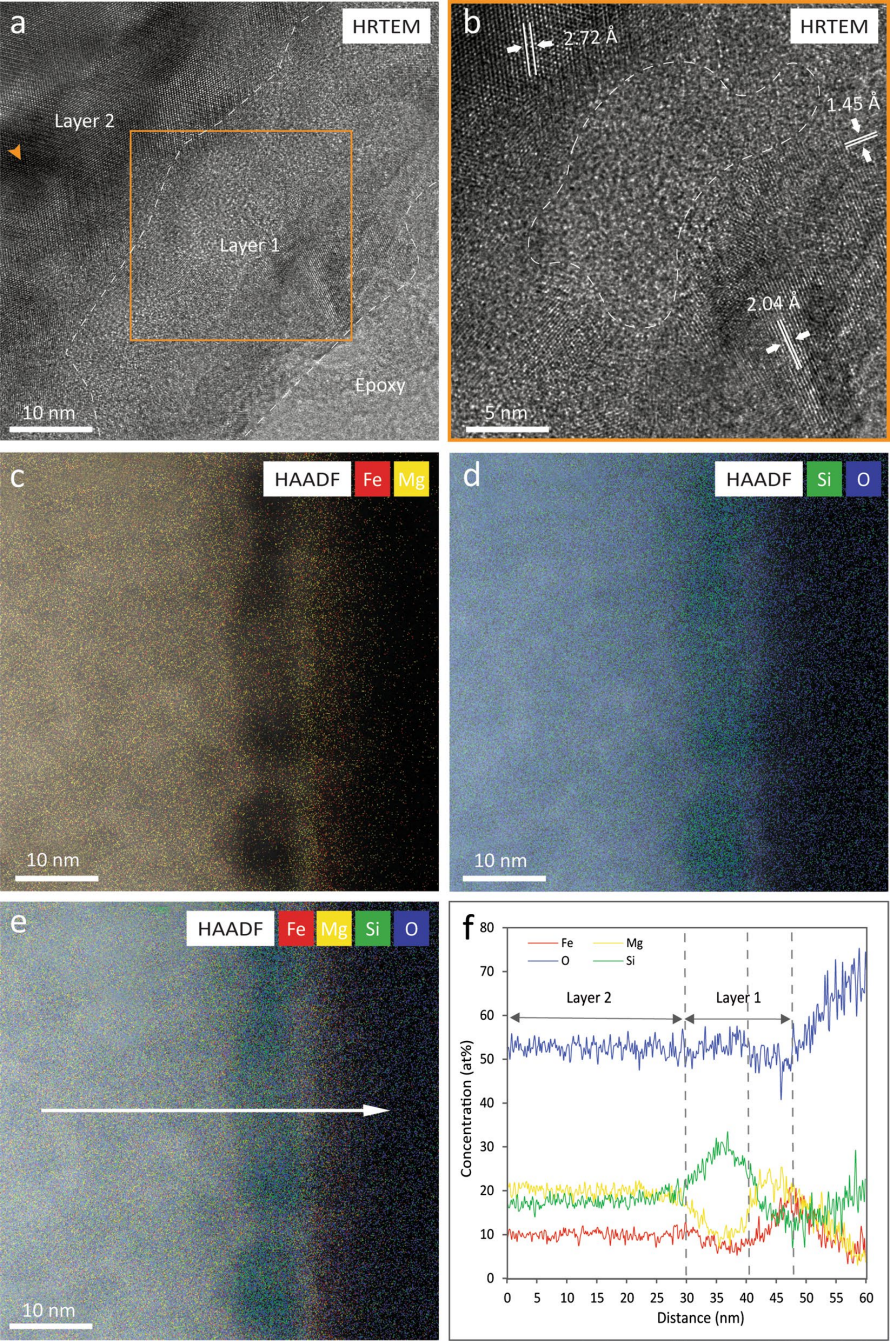
Multilayer rim in olivine grain. **a** HRTEM image exhibiting microstructural layers 1 and 2. **b** HRTEM image showing the occurrence of lattice fringes in layer 2 with d-spacing values of 2.72 Å corresponding to (130) olivine, and in layer 1 values of 2.04 Å and 1.45 Å similar to (110) and (200) metallic iron, respectively. Dashed area within the nanocrystalline region point out the presence of amorphous domains. **c** HAADF image overlain with EDS maps of Fe and Mg. **d** HAADF image with Si and O EDS maps. **e** HAADF image with EDS maps of Fe, Mg, Si, O showing the location of the line scan. **f** Quantitative EDS line scan indicating the presence of three chemically distinct sections

Bright-field transmission electron microscopy (BFTEM) images show the presence of a ~ 50 nm thick discontinuous altered rim on the surface of the olivine grain (Fig. 3). Microstructurally, the rim is divided into two layers: an outer ~ 5–15 nm nanocrystalline layer with minor amorphous material (Layer 1), and an inner ~ 35 to 45  nm thick crystalline layer (Layer 2) (Fig. 4a, b). HRTEM imaging of Layer 2 shows the presence of lattice fringes with d-spacing values of 2.72 Å, similar to (130) olivine. The amorphous Layer 1 presents d-spacing values of 2.04 Å and 1.45 Å, similar to (110) and (200) metallic iron (Fig. 4b). Strained regions were identified as shown by the orange arrow in Fig. 4a. Chemically, the altered rim is divided into three sections: an innermost section that corresponds to the microstructural Layer 2 and presents a similar composition to the unaltered grain, a middle section that is enriched Si but depleted in Mg and Fe compared to the innermost section, and an outer section that is depleted in Si and O but enriched in Fe and Mg compared to the middle section (Fig. 4c–f). The middle and outer sections comprise microstructural Layer 1 identified by HRTEM and the innermost section corresponds to part of the microstructural Layer 2.

Other mineral phases like low-Ca pyroxene and plagioclase were identified in the RC-MD01-0025 Itokawa regolith particle by EDS analysis before allocation. However, we only identified low-Ca pyroxene in the interior of the grain and we did not find any evidence that suggest it was exposed to space weathering. Additionally, we identified plagioclase in the samples and analyzed the material using BFTEM and STEM imaging, and EDS mapping. However, we observed that plagioclase was quickly damaged by the electron beam making it difficult to identify primary space weathering features. Rapid damage of plagioclase grains on Itokawa soil particles during TEM analysis has been previously reported by Noguchi et al. (2011, 2014a).

## Discussion

We identified diverse microstructural and chemical features in pentlandite, troilite, and olivine grains in the Itokawa regolith particle RC-MD01-0025 produced by space weathering processes. These include the presence of crystalline, nanocrystalline, and amorphous rims on the mineral grains, sulfur and nickel depletion accompanied by iron enrichment, and chemically distinct layers. These results provide evidence that sulfide and silicate minerals respond differently to space weathering on the surfaces of airless bodies.

### Space weathered rims

HRTEM analyses demonstrate the presence of rims with different microstructural characteristics in individual mineral phases in the Itokawa regolith particle. Pentlandite has a ~ 5–10 nm crystalline rim with d-spacings similar to pentlandite or Fe-oxides (i.e., magnetite and hematite) while troilite presents a ~ 10 nm nanocrystalline rim. In contrast, olivine presents a ~ 50 nm crystalline rim. No amorphization is observed in the rims of the sulfide minerals which is consistent with analyses of rims on other sulfide minerals from asteroid Itokawa, which were also nanocrystalline (Keller and Berger 2014; Burgess and Stroud 2021). In contrast, rims on olivine grains from Itokawa have previously been reported to present disordered microtextures (i.e., nanocrystallinity surrounded by amorphous domains) and for these layers to extend further into the interior of the mineral grains, up to 50 nm (Keller and Berger 2014). The thicknesses of the altered rims on silicate minerals reported here and in Keller and Berger (2014) and Thompson et al. (2014) are consistent with the penetration depths for 1 keV H^+^ and 4 keV He^+^ (i.e., 27 nm and 51 nm, respectively) estimated by Noguchi et al. 2014a, b using the stopping and range of ions in matter (SRIM) software. This confirms that the thickness of the space weathered rims in these olivine grains from asteroid Itokawa are likely controlled by the penetration depths of the solar wind ions.

Ion irradiation experiments performed on both silicate and sulfide minerals support the findings that the latter are more resistant to amorphization. 1 MeV Kr^2+^ irradiation experiments on pyrrhotite, troilite, and olivine by Christoffersen and Keller (2011) show that Fe-sulfide phases retained their crystallinity after ion irradiation of fluences up to 10^16^ Kr^2+^/cm^2^. While electron diffraction patterns of olivine revealed amorphization after fluences of 10^14^ Kr^2+^/cm^2^, similar measurements for troilite and pyrrhotite revealed no evidence of amorphization after experiencing a maximum dose of 10^16^ Kr^2+^/cm^2^. The resistance of sulfides to amorphization under ion irradiation conditions has been attributed to their low structural complexity (e.g., low number of unique cation sites). Atoms removed from their lattice positions by displacement damage in low-complexity structures are more likely to come to rest in sites that can accommodate the displacement, which would result in a lower probability of generating a defect and drive amorphization (Wang et al. 1991). As sulfide crystal structures are more accommodating, they are able to retain crystallinity more readily in comparison to their silicate counterparts. Additionally, the resistance of sulfides to glass formation due to their low-complexity crystalline structures and their metallic thermal conductivity may also serve as mechanisms to help sulfides recover from displacements that occurred during ion irradiation experiments (Christoffersen and Keller 2011). The combination of these crystal structural characteristics and physical properties of sulfides may also contribute to the resistance of these mineral phases to amorphization under ion irradiation conditions.

### Sulfur depletion

The pentlandite and troilite phases in grain RC-MD01-0025 show evidence for sulfur depletion in their space weathered rims. The sulfur depletion identified in both sulfide phases extends up to ~ 10 nm below the mineral surface. In pentlandite, this S depletion is accompanied by Ni loss, as discussed below. The depth of sulfur depletion is similar to results reported for a pyrrhotite grain by Keller and Berger (2014) and in troilite by Burgess and Stroud (2021). Furthermore, these measurements are supported by ion irradiation experiments that have identified possible mechanisms that could limit sulfur depletion in Fe-sulfide rims to the upper few nanometers. Keller et al. (2010) conducted 4 keV He^+^ irradiation experiments on troilite using total fluences of 10^18^ He^+^/cm^2^, performed TEM and EDS analyses of the irradiated sulfide, and conducted Monte Carlo simulations using the SRIM software to understand the ion–sulfide interactions. In the laboratory experiments, troilite developed a S-depleted and Fe-enriched layer in the outermost ~ 10 nm of the grain. The collisional models indicate that the depth of the maximum ion deposited collisional energy was about ~ 6.5 nm, similar to the extent of sulfur depletion in the experimental samples. In addition, other laboratory simulations of solar wind irradiation of troilite employed X-ray photoelectron microscopy (XPS) to reveal the preferential sputtering of sulfur from the surface of the sulfide during 4 keV He^+^ and Ar^+^ ion irradiation experiments (Loeffler et al. 2008). Moreover, Christoph et al. (2022) identified sulfur depletion after 1 keV H^+^ and 4 keV He^+^ irradiation of troilite from Canyon Diablo and Toluca meteorites using XPS and performed the collisional simulations on the Static and Dynamic Trim for Sequential and Parallel computer (SDTrimSP) software to identify the physical factors contributing to the sulfur depletion. The simulations show that by adding radiation-enhanced diffusion on the model, the fit between the concentrations of Fe and S obtained from SDTrimSP and the concentrations identified in the XPS data improved significantly. During radiation-enhanced diffusion, ion irradiation creates defects in the target lattices (i.e., vacancies and interstitials) (Betz and Wehner 1983) that promote the diffusion of volatile species towards the surface where they are subsequently sputtered (Christoph et al. 2022). This suggests that in addition to sputtering, radiation-enhanced diffusion might enhance sulfur loss in sulfides during ion irradiation from the solar wind.

In contrast to sulfur depletion shown in ion irradiation experiments, pulsed-laser irradiation of troilite performed by Loeffler et al. (2008) showed an increase of the S:Fe ratio from 1.0 in the unirradiated sample to 1.8 in the laser irradiated troilite, attributed to sulfur segregation to the surface of the mineral promoted by heating. Noble et al. (2011) also identified surface sulfur enrichment in pulsed-laser irradiation experiments on ordinary chondrites. Based on the contrasting results obtained in the solar wind irradiation and the micrometeoroid bombardment experiments, and the comparison between our observations and previous studies of sulfides from asteroid Itokawa, we attribute the sulfur depletion identified in pentlandite and troilite from RC-MD01-0025 to preferential sputtering of sulfur caused by solar wind irradiation that might be also enhanced by radiation-damage diffusion processes.

In addition, we identified strained regions in both the sulfide and olivine grains as shown in Figs. 1.b, 2.b, and 4a. Strain corresponds to modifications in lattice spacings caused by structural defects or changes in chemistry (Williams and Carter 2009). These strained regions on sulfides could have been produced by implantation of solar wind ions as similar features have been identified in an He^+^ irradiated olivine by Laczniak et al. (2021).

### Ni depletion

Nickel depletion was identified in the crystalline rim of the pentlandite grain. The extent of Ni loss is consistent with the depth of sulfur depletion and iron enrichment suggesting that the same mechanisms resulting in changes in S and Fe concentration might also be driving Ni depletion. Preliminary observations of a pentlandite grain in samples returned from asteroid Ryugu by the Hayabusa2 mission show the formation of iron whiskers on the surface of the sulfide grain that are depleted in S and only contain small amounts of Ni (Matsumoto et al. 2022). Similar Fe-rich and S-depleted whiskers have been identified in Fe-sulfides from asteroid Itokawa and lunar mare soils (Matsumoto et al. 2020, 2021). As S depletion in Fe-sulfides has been reported in both returned samples and laboratory simulations and has been attributed to space weathering processes, the identification of rims in this study and whiskers depleted in S and Ni (Matsumoto et al. 2022) in Fe–Ni-sulfides from asteroidal regoliths suggest that space weathering might promote Ni loss in Ni-bearing sulfides.

The development of Ni- and S-depleted rims in pentlandite could result from many factors including differences in binding energy for the individual elements and/or bond strength in the crystal structure. Preferential sputtering of S in sulfides occurs because this element has a low binding energy. However, Fe and Ni in sulfide minerals have much higher binding energies compared to sulfur indicating that it is unlikely these species would be preferentially sputtered from the pentlandite grain based on binding energy alone (Legrand et al. 2005). However, there are differences in bond strength when comparing Ni–S and Fe–S bonds. Ni–S bonds are stronger compared to Fe–S in pentlandite as they have a shorter bond length (Rajamani and Prewitt 1975). Consequently, it is possible that during solar wind ion irradiation, Ni–S is sputtered as a molecule, rather than individual atoms from the pentlandite surface, ultimately forming a Ni- and S-depleted rim that is enriched in Fe. This is supported by the coincident depth of depletion for both Ni and S in the sample rims. In addition, ion irradiation experiments on metals (Laegreid and Wehner 1961; Rosenberg and Wehner 1962; Chuang and Wandelt 1978) identified slightly higher sputtering yields of Ni compared to Fe which could also promote a preferential loss of Ni over Fe in the rim. Considering these results together, the formation of the rim in pentlandite results from solar wind irradiation as we do not see any clear evidence of the influence of micrometeoroid impacts in the microstructural and chemical properties of troilite, pentlandite, and olivine. Experimental efforts are underway to understand the response of pentlandite under space weathering conditions.

The identification of nickel depletion in a pentlandite grain from asteroid Itokawa in this study corresponds to the first report of nickel depletion in a rim as result of space weathering. These results may help us identify space weathering signatures in pentlandite grains from asteroid Ryugu, as this mineral has been identified in the returned regolith samples (Han et al. 2022; Matsumoto et al. 2022; Viennet et al. 2022). Additionally, it is likely that pentlandite is present in the returned regolith particles from asteroid Bennu, as this is a common mineral phase in CM chondrites (Kimura et al. 2011) that are the closest compositional match for Bennu (Hamilton et al. 2019).

Moreover, in this study and similar to the results reported by Keller and Berger (2014), we did not identify the presence of iron whiskers protruding from the surfaces of the sulfide grains in contrast to Matsumoto et al. (2020, 2021). The absence of whisker in both studies could correspond to differences in exposure times. However, it is still not clear how other processes such as diffusion and heating produced by micrometeoroid impacts could contribute to the development of this iron whiskers on the surfaces of Fe-bearing sulfides.

### Chemically heterogeneous layers

Olivine presents chemically distinct layers in the ~ 50 nm altered rim. The rim presents three different sections, an inner section that exhibits the same composition as the unaltered grain, a middle section that is enriched in Si and depleted in Fe and Mg compared to the inner region, and an outer section that is enriched in Fe and Mg and depleted in Si and O compared to the middle section. The middle and outer sections correspond to the nanocrystalline Layer 1 identified by HRTEM imaging.

Similar chemically heterogeneous rims have been previously identified in olivine grains from asteroid Itokawa by Noguchi et al. (2011, 2014a), although the outermost layer described in those studies is not only enriched in Fe and in Mg as in our analyses of the olivine in RC-MD-01–0025, but is also enriched in elements that are exogenous including Cl, K, Na, and Ca. Noguchi et al. (2014a) describes this outermost layer as be the result of redeposition of sputtered ions by solar wind irradiation or vapor deposition caused by heating from nearby micrometeoroid impacts. However, EDS analysis did not show the occurrence of exogenous elements in the outer section on the olivine grain analyzed in this study. Moreover, Thompson et al. (2014) reported a similar chemically distinct layered rim in a pyroxene grain from asteroid Itokawa. The Si-rich middle section in the pyroxene grain is nanocrystalline and the Fe- and Mg-enriched in the outer sections is amorphous. However, we observe nanocrystallinity in the Fe- and Mg-enriched and the Si-rich sections in the olivine grain compared to the findings in Thompson et al. (2014)

The identification of chemically distinct layers in silicates as result of space weathering processes has not been limited to returned samples, but has been also observed in solar wind irradiation experiments. Laczniak et al. (2021) conducted 1 keV H^+^ and 4 keV He^+^ irradiation experiments on a Murchison chip and performed coordinated analysis to characterize the microstructure and chemistry of the irradiated meteorite. TEM and EDS observations of an H^+^ irradiated Fe-rich olivine grain show the occurrence of three different zones in an altered rim developed after irradiation. The outer ~ 2 to 5 nm thick region in those samples is mostly amorphous and enriched in Fe and Mg, the middle region is Si-rich and is completely amorphous, and the innermost region corresponds to a combination of amorphous and crystalline material that presents the same chemical composition as the unaltered olivine. Several mechanisms have been proposed as the driving factors forming these chemically distinct layers identified in both returned samples and laboratory simulations, including sputtering, radiation-enhanced diffusion, impact vapor deposits, sputtered redeposition, and recoil mixing. However, as the chemical layers identified in the ion irradiation experiments by Lazcniak et al. (2021) are similar to the chemically distinct layers that we identified in the olivine grain in this study, it is most likely that solar wind irradiation is the main factor driving these chemical signatures in samples from Itokawa. Additionally, we did not observe any microstructural evidence (e.g., melt deposits) in the regolith particle that supports that micrometeoroid bombardment might have influenced the space weathering features in the RC-MD01-0025 regolith particle. Therefore, we will focus on the possible mechanisms associated with ion irradiation that might have promoted the formation of these chemical layers in the olivine grain.

The development of chemically heterogeneous layers in olivine is likely explained by a combination of multiple mechanisms. The observed segregation of Si, Mg, Fe, and O in the space weathered rim is not directly correlated to atomic mass, binding energy or stoichiometric relationships. Instead, we suggest radiation-enhanced diffusion and sputtering as possible mechanisms controlling the formation of the chemically distinct layers in olivine. Studies focused on the influence of H^+^ in diffusion of Fe and Mg in olivine identified a direct dependence of the diffusion rate of these elements on the concentration of cation vacancies in the olivine structure, with the diffusion of Fe and Mg in olivine being enhanced under hydrous conditions compared to an anhydrous setting. This increased diffusion rate was facilitated by the higher concentration of vacancies formed by the incorporation of the H^+^ in the olivine (Wang et al. 2004). Additionally, previous studies regarding diffusion rates of Fe, Mg, Si, and O in olivine show that diffusion rates of Fe and Mg are faster compared to Si and O (Hermeling and Schmalzried 1984; Chakraborty et al. 1994; Dohmen et al. 2002). These results suggest that radiation-enhanced diffusion caused by cation vacancies might promote the migration of Fe and Mg towards the surface of the olivine grain forming the outer Fe- and Mg-rich layer. In addition to the contribution of radiation-enhanced diffusion to the development of the chemically heterogeneous layers, sputtering could explain the O depletion of the outermost region and the formation of metallic iron-rich nanocrystalline layer.

Sputtering depends on several factors, including binding energies, mass of the atoms, angle of incidence, and elemental abundances in the target. Dukes et al. (1999) identified a reduction of the oxidation state of Fe^3+^, depletion of O, and the formation of npFe^0^ during He^+^ irradiation in olivine. These observations were attributed to the influence of binding energies during sputtering as Fe–O bonds are weaker than Si–O and Mg–O bonds (Reed 1971; Liebau 1985; Weast 1985) and therefore are easier to disaggregate during sputtering. This suggests that O is preferentially sputtered from Fe–O in olivine by solar wind ions, promoting the development of an outermost section depleted in O and the formation of metallic iron nanoparticles in the Itokawa olivine grain. The chemically heterogeneous rims present in pyroxene (Thompson et al. 2014) and olivine (this study) grains from asteroid Itokawa and their similarity with ion irradiation experiments (Laczniak et al. 2021) suggest that solar wind irradiation is driving the formation of these rims on silicates in airless bodies.

In addition, we did not observe blistering on the surface of the olivine grain in contrast to the vesicular rims identified in Itokawa olivine and pyroxene grains by Noguchi et al. (2014a) that have been attributed to implantation of solar wind ions. The microstructure of the space-weathered rim in olivine in this study is similar to the properties of olivine analyzed by Noguchi et al. (2014b) and Keller and Berger (2014). The discrepancies could correspond to a difference in the stage of space weathering. However, there is uncertainty in the exposure times as solar flare tracks were not observed in this study nor in Keller and Berger 2014.

## Conclusion

We investigated the chemical and microstructural properties of space-weathered pentlandite, troilite, and olivine grains from a regolith particle from asteroid Itokawa. Microstructurally, the sulfide grains and olivine present crystalline and nanocrystalline rims. The space-weathered rims in pentlandite and troilite are both S-depleted and enriched in Fe, and the pentlandite rim is also depleted in Ni. The altered rim in olivine is divided in three sections, and inner section that has the same compositional characteristics as the unaltered region, a middle section enriched in Si, and an outer section enriched in Fe and Mg. We attribute the development of these microstructural and chemical signatures to a dominant influence of solar wind irradiation. The differences in the space weathering signatures of both mineral phases indicate that sulfides and silicates respond distinctly to similar conditions in the interplanetary space. Studying the response of sulfide minerals to space weathering conditions through returned sample analysis and laboratory simulations is crucial to understand how planetary bodies like S-type and C-type are altered under micrometeoroid impacts and solar wind irradiation. Identifying the microstructural and chemical characteristics produced by space weathering in sulfides will help us perform a more accurate characterization of the returned samples from asteroids Ryugu and Bennu.

## Data Availability

Data available upon request.

## Abbreviations

SEM: Scanning electron microscopy
TEM: Transmission electron microscopy
EDS: Energy-dispersive X-ray spectroscopy
HRTEM: High-resolution transmission electron microscopy
BFTEM: Bright-field transmission electron microscopy
STEM: Scanning transmission electron microscopy
HAADF: High-angle annular dark field
JAXA: Japan Aerospace Exploration Agency
